# Assessing and quantifying the interactions between spasticity, proprioception, and motor function of the upper limb after stroke: A meta-analysis

**DOI:** 10.1177/20556683251372085

**Published:** 2025-09-17

**Authors:** Jasmine Usher, Jacqui Morris, Alejandra Aranceta-Garza

**Affiliations:** 1 Biomedical Engineering, School of Science and Engineering, 3042 University of Dundee, UK; 2 Centre for Medical Engineering and Technology, 3042 University of Dundee, UK; 3 School of Health Sciences, 3042 University of Dundee, UK

**Keywords:** stroke, spasticity, proprioception, motor function, upper limb

## Abstract

**Objective:**

The objective of this study was to carry out a systematic review and meta-analysis of available literature on the relationships between spasticity, proprioception and motor function of the upper limb post-stroke.

**Methods:**

Using the terms: stroke; movement; proprioception; spasticity; rehabilitation; and upper limb, a systematic search was conducted on Scopus, PubMed and Web of Science from database inception to November 2023. A study must have assessed two of spasticity, proprioception, or motor function of the upper limb post-stroke to be included. Random-effects meta-analyses were conducted to investigate changes in time and strength of correlations between variables.

**Results:**

Fifty-two studies were included. Over time, spasticity increased (OR = 0.5, p = 0.0475); proprioception and motor function impairments decreased (OR = 3.15, *p* < 0.0001; OR = 3.21, *p* < 0.0001, respectively). The correlation between spasticity and proprioception was weak (r = 0.33, *p* = 0.0283); between proprioception and motor function was moderate (r = 0.45, *p* < 0.0001); and between spasticity and motor function was moderate (r = 0.55, *p* < 0.0001).

**Conclusion:**

Despite the limitation of heterogeneity in the available evidence, relationships between variables were illustrated. Moderate correlations between proprioception and both spasticity and motor function suggest proprioception should be an important target for personalised rehabilitation interventions.

## Introduction

Stroke is the second leading cause of mortality worldwide, surpassed only by heart disease.^1^ Notably, 15% of strokes occur in people under the age of 50^2^. In 2022, approximately 101 million people across the world were living with the aftermath of stroke, and with around 12.2 million new strokes per year, this figure will continue to rise.^2^ Strokes are classified into two types: ischemic which make up 85% of all cases and involve a loss of blood flow to the brain caused by the presence of a blood clot, and haemorrhagic strokes, caused by bleeding in the brain.^2^ An acute stroke lesion consists of a dead neuron core surrounded by a penumbra of dysfunctional neurons. Depending on its location within the brain, this lesion can lead to changes in neural functioning, causing impairments that negatively impact functional performance and independence in activities of daily living.^3^

Spasticity, defined as a velocity-dependent increase in stretch reflexes,^4^ is a common post-stroke complication.^5^ These stretch reflexes are a function of the intrafusal, muscle spindles within skeletal muscle, which detect changes in muscle length. When these spindles detect rapid length changes, they trigger reflexes that oppose the movement to prevent muscle damage.^5^ Following a stroke, lesions that affect the descending tracts of supraspinal origin can disrupt the balance of inhibitory and facilitatory inputs to the spinal cord. This disruption leads to stretch reflex hyperexcitability, resulting in spasticity.^6^

Stroke lesions can lead to various other modifications in neural function. For example, lesions affecting areas of the brain involved in the processing of somatosensory information can cause sensory function transformations. This can include changes in proprioception,^7^ the body’s ability to sense movement, position and resistance applied to its extremities.^8^ Proprioception relies on specialized sensory organs including Golgi tendon organs, muscle spindles and other sensory receptors present in the skin and joints.^8^ Muscle spindles are regarded as the primary proprioceptors^8^ making them integral to the proprioception system.

Both the development of spasticity and changes in proprioception function can cause difficulty in movement^5^ and hinder a person’s ability to perform everyday activities.^9^ Additionally, contracture - the permanent loss of range of motion in passive joint movement - often develops through disuse of the muscles affected by spasticity and can cause further functional problems.^10^

Despite the known impact of both spasticity and proprioception on motor function impairments, there is limited research into their combined effect on rehabilitation outcomes. The involvement of muscle spindles within both stretch reflexes and proprioception suggests a potential underlying association between spasticity and proprioception that could be leveraged in stroke rehabilitation. To develop highly effective rehabilitation programs, a deeper understanding of relationships between spasticity, proprioception and motor function is required. This understanding would allow changes in one function to predict possible changes in others enabling rehabilitation to be tailored to individual’s abilities and likely functional progression. Therefore, this work aims to investigate current published evidence on the relationships between spasticity, proprioception, and motor function of the upper limb post-stroke. This investigation will provide a more comprehensive understanding of existing interactions and recommend potential future research areas for stroke recovery and rehabilitation.

## Methods

This research involved the use of systematic review and meta-analysis methodologies.

### Study design

Throughout this work, the PRISMA 2020 guideline was followed.^11^ A protocol for review methods was reviewed and agreed upon by all authors before implementation. One author completed all search, screening, critical appraisal, data extraction, and statistical analysis processes. If there was doubt about the inclusion of a paper or the use of a particular process, this was discussed between all authors until consensus was achieved.

### Literature search

A systematic search was completed on Scopus, PubMed, and Web of Science from database inception to November 2023. The keywords used were: stroke; movement; proprioception; spasticity; rehabilitation; upper limb and their synonyms.

### Selection of studies

Studies were included if they assessed at least two of the following: spasticity, proprioception, or motor function of the upper limb after stroke, and if they were fully available in English.

Studies were excluded if they met any of the following criteria:

• Were reviews or meta-analyses
• Were not based on stroke
• Did not examine the upper limb
• Failed to provide statistical analysis between at least two of the variables of interest
• Failed to provide raw data that would allow the authors to perform between-variable analysis

No studies were excluded based on the measurement tools used for each variable.

Once studies were assessed for inclusion, quality appraisal was conducted using the Mixed Methods Appraisal Tool.^12^

### Data extraction

From all studies eligible for inclusion the following data, if reported by a study, was extracted: the sampled population per study; participant age; time since stroke at the start of the study; sex; the type of stroke (including the population percentage within a study that had a first-ever stroke); the measurement tool used for assessment of each variable (spasticity, proprioception and motor function); between variable relationships assessed; number and timing of multiple assessment timepoints and if anti-spasticity medications were prescribed to participants.

### Data analysis

Data analysis was split into two sections: an analysis of the included studies’ characteristics, followed by meta-analyses of the changes in variables of interest (spasticity, proprioception and motor function) over time and the relationships between these variables.

#### Characteristics of included studies

To examine the characteristics of the included studies, extracted data were tabulated. To homogenize continuous data, summary statistics were converted to means (as described by Hozo et al.^13^ or Luo et al.^14^). Where the means of two subgroups (with and without spasticity, for example) were reported, a combined mean was calculated. Categorical data were reported as number or percentage of sample. Analysis of data describing study characteristics was conducted using the Statistical Package for the Social Sciences (SPSS) version 29.

#### Quantitative synthesis

Random-effects meta-analyses were then carried out using R version 3.4.2 to investigate: (i) changes of variables (spasticity, proprioception and motor function) in time (where different timepoint data was provided); and (ii) strength of relationships, if any, between variables. The random-effects model was used to account for heterogeneity in population characteristics and measurement tools used between studies. Heterogeneity was assessed using the I^2^ statistic and defined as: [0%–24%]: negligible, [25%–49%]: low, [50%–74%]: moderate and [75%–100%]: substantial.^15^ Statistical significance was set at α = 0.05 for all analyses. Egger’s regression test was used to assess the publication bias of analyses including more than 10 studies.^16^

##### Changes in time for each assessed variable

Studies that provided individual participant data at different timepoints were identified and changes in time for each variable (spasticity, proprioception, and motor function) were quantified as follows: the presence of spasticity, impaired proprioception and impaired motor function in participants were treated as dichotomous variables (present or not present) at the first and final assessment timepoints within a study (defined as *start* and *end*). The effect estimate used for the meta-analysis was Odds Ratio (OR) using a continuity correction of 0.5 for zero-frequency entries.

##### Strength of relationships between variables

The relationships between variables were explored as (a) spasticity vs proprioception; (b) proprioception vs motor function; and (c) spasticity versus motor function. Random-effects models were used to pool correlation coefficients of these relationships from all studies which provided these. Additional correlations were estimated (two-tailed Pearson or Spearman, as appropriate) from one study (Franck et al.^17^) where data was available, but correlations were not provided.

All correlation coefficients for each relationship, regardless of their statistical significance and the methods used to obtain them were included in the meta-analysis. If a study reported multiple correlations between variables, for example, if different motor function measurement tools (e.g. the Fugl-Meyer assessment and the Action Research Arm Test) were used and both used to calculate a correlation between spasticity and motor function, a mean correlation coefficient was calculated, and it was this coefficient used in the analysis. Only the strength of the correlations was of interest, so the absolute values of reported and calculated coefficients were used in the analysis. Fisher’s z transformation of correlation coefficients was used to achieve a normal distribution before analysis.

## Results

**Figure 1.**
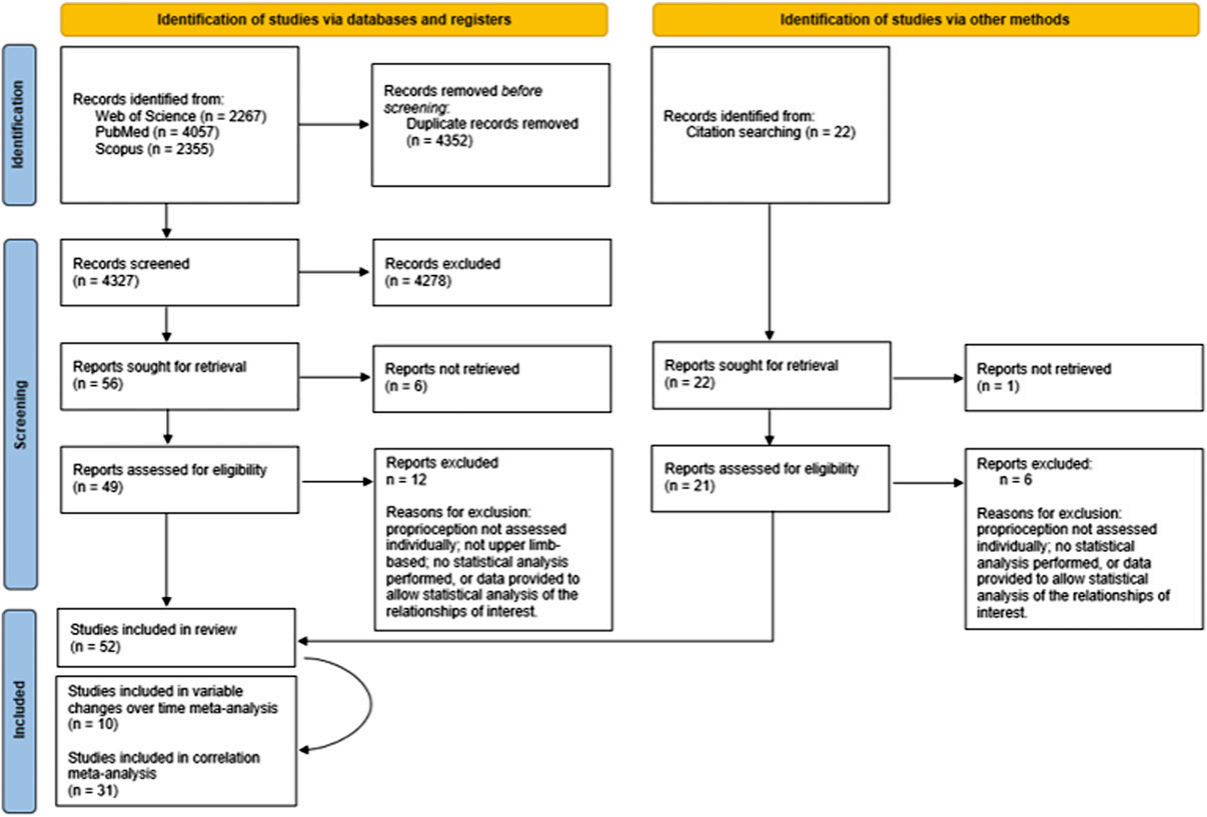
PRISMA flow diagram indicating a start of 8701 records being identified, with a total of 8649 excluded at different stages leading to a total of 52 included in this review, of which 10 were included in the meta-analysis of changes in variables over time and 31 were included in the correlation meta-analysis. Note: two studies were included in the change of time analysis and the correlation meta-analysis. The remaining 13 studies did not provide enough information to be included in any meta-analysis but are still included in the review section of this work. In the reasons for exclusion, ‘proprioception not assessed individually’ indicates proprioception was grouped with other sensory functions rather than reported on its own.

A total of 8701 studies were initially identified, with 52 eligible for inclusion (Figure 1, the PRISMA flow diagram is shown). The included studies consisted of longitudinal studies, observational studies and clinical trials of upper limb functions.

### Characteristics of included studies

#### Sampled population

The sampled population of each study ranged from 10 to 3056 participants totaling 6407 participants across the 52 studies. In total, 10 studies (19.23%) included ≤20 participants; 28 (53.84%) included ≥20 and ≤100 participants; 12 (23.07%) included ≥100 and ≤200 participants; and three (5.76%) studies included ≥200 participants (see Table 1).

**Table 1.** Demographic data for each of the 52 studies included in this review divided by number of participants; age at the start of the study; time since stroke at the start of the study; sex; % of sample with ischemic stroke; relationship(s) assessed; % of sample with first ever stroke; if follow-up sessions occurred (yes/no); when after the start of the study those follow-ups occurred; if participants were taking anti-spasticity medication throughout the study period (yes/no) including what type of medication was given. M = male; F = female; S = spasticity; P = proprioception; and MF = motor function. If the relationship between spasticity and proprioception was assessed it is noted as “SvsP”, between proprioception and motor function as “PvsMF” and between spasticity and motor function as “SvsMF”. BA = baclofen; BE = benzodiazepine, BO = botulinum toxin, EX = excluded if taking anti-spasticity medication. ‘-’ indicates no data available or non-applicable.

Reference	Sample size	Mean age at the start (years)	Mean time since stroke at the start of study (months)	Sex (M/F)	% Of ischemic stroke	Relationship/s assessed	% Of the sample with first-ever stroke	Multiple assessment points? (Y/N)	Time since start of study at follow-up assessments (months)	Anti-spasticity medication? (Y/N)
Mochizuki et al.^17^	70	59.7	10.5	49/21	-	SvsP; SvsMF	-	N	-	Y (BA; BE)
Pundik, Falchook, McCabe et al.^18^	23	56.3	21.6	14/9	88.6	SvsP; SvsMF	-	N	-	N
Wagner et al.^19^ (2006)	46	63.9	0.31	18/28	-	SvsP; PvsMF; SvsMF	-	N	-	N
Dukelow et al.^20^	100	62.9	0.94	57/43	83.0	PvsMF	-	N	-	N
Meyer et al.^21^	122	67.3	3.04	77/45	88.5	PvsMF	100	N	-	N
Welmer et al.^22^	66	76.0	0.16	22/44	-	PvsMF	100	Y	3; 18	N
Cherpin et al.^23^	20	55.7	13.2	12/8	-	PvsMF	100	Y	0.75; 1.5; 3; 6	N
Arya et al.^24^	95	48.6	6.73	80/15	81.1	PvsMF	100	N	-	N
Rand^25^	102	59.6	20.9	69/33	-	PvsMF	-	N	-	N
Rand et al.^26^	40	73.4	0.57	17/23	-	PvsMF	-	Y	0.5; 1; 1.5	N
Rand, Weiss and Gottlieb^27^	20	72.5	0.60	7/13	-	PvsMF	-	Y	0.5; 1; 1.5	N
Wagner et al.^28^ (2007)	39	63.9	0.29	15/24	-	PvsMF; SvsMF	-	Y	6	N
Bourke et al.^29^	38	63.5	0.92	21/17	97.3	PvsMF	-	N	-	N
Najafabadi et al.^30^	225	56.5	41.0	113/112	74.4	PvsMF	-	N	-	N
Semrau et al.^31^	116	-	0.35	-	-	PvsMF	-	Y	1.5; 3; 6.5	N
Huang et al.^32^	57	62.0	0.66	34/23	73.7	PvsMF; SvsMF	100	N	-	N
Franck et al.^33^	10	56.3	1.18	10/0	90	SvsMF	100	Y	0.25; 0.5; 0.75; 1; 1.25; 1.5; 1.75; 2; 2.25; 2.5; 2.75; 3; 3.5; 4; 4.5; 5; 5.5; 6	Y (BO)
Kong et al.^34^ (2010)	140	61.1	41.7	88/52	72.8	SvsMF	100	N	-	N
Kong et al.^35^ (2011)	140	61.1	41.7	88/52	72.8	SvsMF	-	N	-	N
Lackritz et al.^36^	42	53.0	2.09	28/14	-	SvsMF	-	N	-	N
Leonard et al.^37^	13	62.8	97.2	10/3	-	SvsMF	-	N	-	N (EX)
Li et al.^38^	28	57.8	76.0	16/12	-	SvsMF	-	N	-	N (EX)
Lin and Sabbahi^39^	10	59.0	48.0	9/1	60.0	SvsMF	-	N	-	N (EX)
Plantin et al.^40^	61	53.0	0.80	43/18	67.0	SvsMF	100	Y	3; 6	Y (BO)
Rech et al.^41^	34	58.4	24.7	23/11	82.4	SvsMF		N	-	N
Ryu et al.^42^	245	61.5	-	133/112	59.2	SvsMF		N	-	N
Sommerfeld et al.^43^	95	78.0	0.18	60/35	-	SvsMF	-	Y	3	N
Thielman et al.^44^	11	56.5	92.8	7/4	-	SvsMF	-	N	-	N
van Kuijk et al.^45^	40	68.0	-	20/20	100.0	SvsMF	60	Y	0.25; 0.5. 0.75, 1.5; 3; 6.5	N
Welmer, von Arbin and Holmqvist et al.^46^	66	76.0	-	22/44	80.3	SvsMF	100	Y	18	Y (BO)
Woldag et al.^47^	10	44.9	34.5	7/3	70.0	SvsMF	-	Y	1; 2; 3	Y (BO)
Davidowitz et al.^48^	16	57.4	-	13/3	87.5	SvsMF	100	N	-	N
Barry et al.^49^	95	68.3	76.8	59/36	-	SvsMF	-	N	-	N
Andringa et al.^50^	17	62.0	-	11/6	100.0	SvsMF	100	Y	1.25; 2; 6.5	Y (BO)
Opheim et al.^51^	117	67.2	0.10	70/47	82.0	SvsMF	100	Y	0.33; 1; 12	Y (BO)
Cacho et al.^52^	27	49.5	41.2	16/11	40.7	SvsMF	-	N	-	N
Harris and Eng^53^	93	-	61.2	-	36.6	SvsMF	-	N	-	N
Huang, Hsieh and Wang et al.^54^	66	53.2	22.6	45/21	59.1	SvsMF	-	N	-	N
Kong, Lee and Chu^55^	163	63.8	-	111/52	100.0	SvsMF	-	N	-	N
Lee et al.^56^	43	57.9	15.2	23/20	67.4	SvsMF	100	N	-	N
Leem et al.^57^	48	58.5	-	30/18	37.5	SvsMF	-	Y	1	N
Levin^58^	10	45.5	27.6	7/3	-	SvsMF	-	N	-	N
Opheim and Danielsson et al.^59^	117	69.3	0.10	66/51	85.0	SvsMF	100	Y	0.33; 1; 3; 6; 12	N
Shin et al.^60^	3056	63.1	-	1781/1275	-	SvsMF	100	Y	3; 6; 12	N
Zackowski et al.^61^	18	55.0	32.0	11/7	77.8	SvsMF	-	N	-	N
Katz et al.^62^	10	-	-	-	-	SvsMF	-	Y	0.25, 0.5	N (EX)
Ada et al.^63^	27	63.0	-	20/7	-	SvsMF	-	Y	0.5; 1; 1.5; 2.25; 4.25; 6.5; 9.75; 12	N
de Jong et al.^64^	50	70.3	-	21/29	100.0	SvsMF	-	Y	0.33-0.4; 3; 6	N
Mirbagheri et al.^65^	21	64.0	-	11/10	33.3	SvsMF	100	Y	1; 2; 3; 6; 12	N
Lin, Huang, and Hsieh et al.^66^	57	55.1	12.0	39/18	-	SvsMF	100	Y	0.75	N
Abdullhai et al.^67^	144	58.7	9.10	88/56	52.1	SvsMF	-	N	-	N
Pundik, McCabe and Skelly et al.^68^	128	59.0	22.1	90/38	-	SvsMF	-	N	-	N
Mean total	124 (total: 6407)	61.02	22.57	75/52	76.7%	11x PvsMF	4105 people with first-ever stroke	22 with multiple assessment points	Mean number of assessment sessions: 4	6x BO 1x BA; BE 4 x EX
						36x SvsMF				
						2x SvsP; SvsMF				
						2x SvsMF; PvsMF				
						1x SvsP; SvsMF; PvsMF				

#### Age

From the 52 included studies, 49 (94.23%) reported data related to age, with a combined mean age of 61 ± 7.41 years, range: [44.9, 76.0].

#### Time since stroke

From the 40 (76.92%) studies that reported on this, the combined mean time after stroke at the start of the study was 23 ± 27.0 months, range: [0.10, 97.2].

#### Sex

Across the 49 (94.23%) studies that provided information about the sex split of participants, 3487 participants were male and 2485 were female (overall 1.4 times more males than females). At an individual study level, the ratio of females over males was [0.000 to 0.430] in 11 studies; [0.430 to 0.860] in 27; [0.860 to 1.290] and [1.290 to 1.720] in four studies each, and [1.720 to 2.150] in three.

#### Type of stroke

Only ischemic and hemorrhagic strokes were studied, with a total of 30 studies (57.69%) providing this information. Of these, 17 (32.69%) included only participants who had sustained a first-ever stroke. Across these studies, a total of 1778 participants presented with ischemic stroke and 577 participants with hemorrhagic stroke.

#### Type of measurement tool used

**Figure 2.**
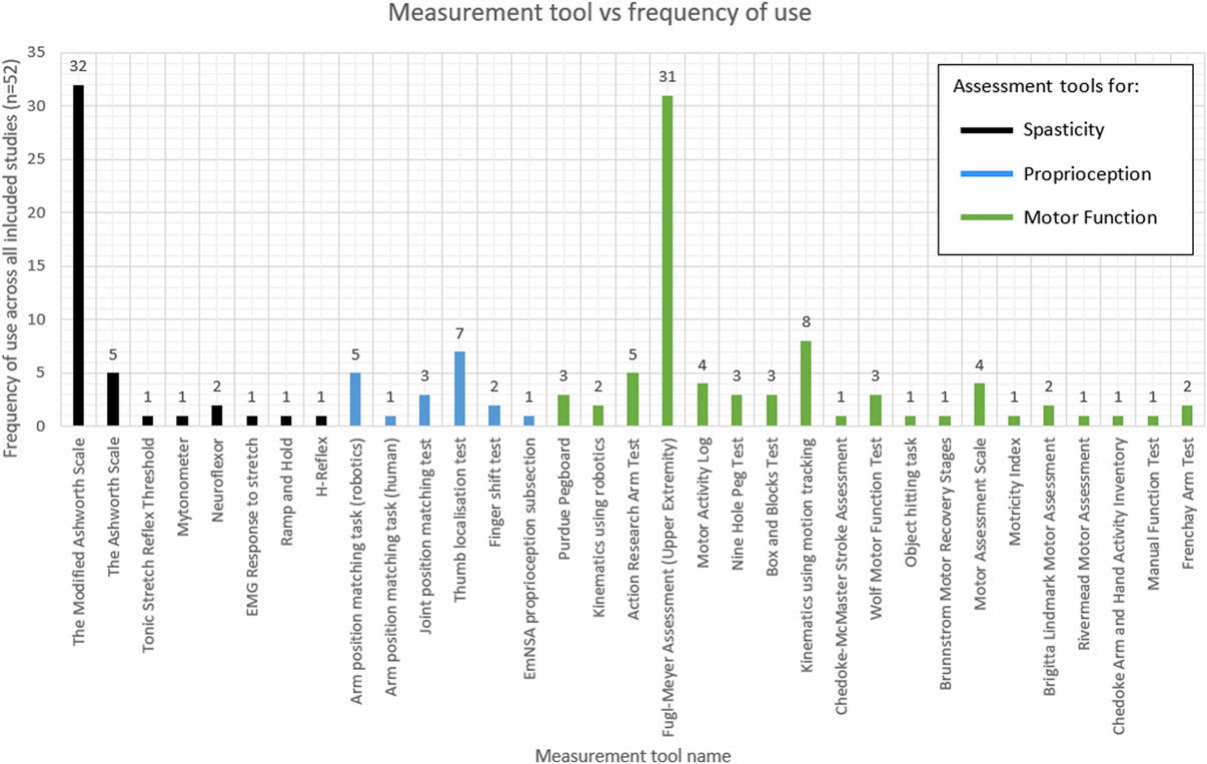
Histogram depicting the frequency of the measurement tools used by variable (spasticity, proprioception and motor function) across included studies (n=52).

Across all 52 included studies, there were eight different spasticity; six proprioception; and 19 motor function measurement tools used. From these, The Modified Ashworth Scale (MAS); Thumb Localisation Test (TLT); and Fugl-Meyer Assessment of the Upper Extremity (FMA-UE) were the most common, respectively. The type and frequency of the assessment tools used across studies are shown in Figure 2.

#### Relationships between variables of interest assessed across included studies

The relationship investigated varied widely across studies. From the 52 studies, five (9.62%) assessed more than one relationship: two assessed the relationship between spasticity and proprioception, and spasticity and motor function; two more assessed proprioception and motor function, and spasticity and motor function; and one assessed all three relationships (spasticity and proprioception, proprioception and motor function and spasticity and motor function). The relationship between spasticity and motor function was assessed individually in 36 (69.23%) studies and the relationship between proprioception and motor function was assessed individually in 11 (21.15%) studies. No study performed a multiple correlation analysis looking into the three variables together.

#### Multiple assessment timepoints

The number of assessments each study used ranged from one to 18, with an average of four sessions across studies. The average time for a final follow-up was 7 months after commencement of the study, range: [0.75 to 18] months.

#### Anti-spasticity medication

There were only seven studies that provided information regarding any anti-spasticity prescription. Amongst those reported were *baclofen*, *benzodiazepines*, and *botulinum*-*toxin*. Participants taking anti-spasticity medication were explicitly excluded in four studies, the rest of the studies did not report anything regarding anti-spasticity medication. There was insufficient evidence to perform sub-group analyses of improvements in those taking medication compared to those not taking medication.

### Quantitative synthesis

Due to the different sampling populations, relationships studied, and details of statistical analyses provided across the different studies, sub-category analyses were not possible. Based on the assessment of multiple variables or relationships, some studies were included in more than one analysis.

#### Changes in time for each assessed variable

All studies in these analyses reported to have followed usual/standard care. No interventions were used nor changes to the standard of care reported. None of the following analyses included enough studies for Egger’s regression to be performed.

##### Spasticity

**Figure 3.**
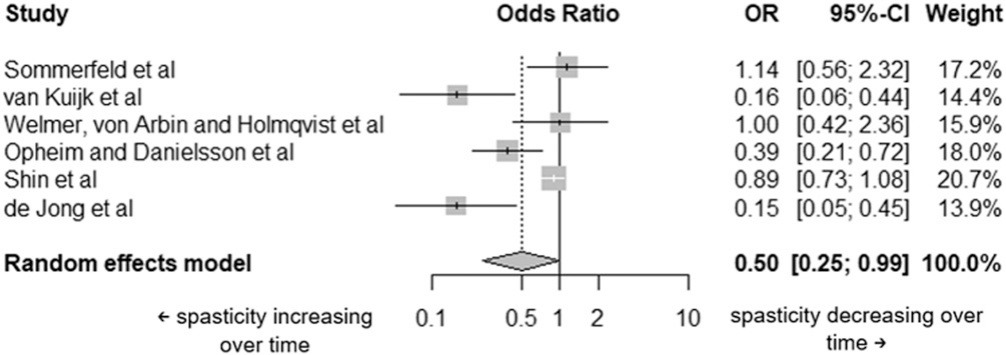
Forest plot of the individual studies included in the spasticity meta-analysis and the overall pooled For Peer Review effect.

As seen in Table 1, from those studies evaluating spasticity (41), 16 provided information from different timepoints. From these, only six studies (3424 participants) provided a sufficient level of detail in their data to understand how spasticity changed per participant over time. Measurement tools used across the six studies were: the Ashworth and Modified Ashworth Scales (in one and five studies, respectively). Spasticity decreased in time between 1 week and 3 months post-stroke in one of these studies (*n* = 95, OR = 1.14 [0.56; 2.32]); two reported little to no change (n = 3056, OR = 0.89 [0.73; 1.08]; and n = 66, OR = 1.00 [0.42; 2.36], respectively) between the start and end of the study (both had an initial assessment at 3 months post-stroke and final assessment at 12 and 18 months post-stroke, respectively); three studies saw an increase of spasticity in time with initial assessments within 1 week of stroke and final assessments at 3 months post-stroke (*n* = 40, OR = 0.16 [0.06; 0.44]; *n* = 113, OR = 0.39 [0.21; 0.72]; and *n* = 50, OR = 0.15 [0.05; 0.45]). The random-effects model ( $I^{2}$ = 80.9%, 95% CI: 58.8%–91.1%) demonstrated that the presence of spasticity at the start is half that of what it is at the end i.e, spasticity increases over time (OR = 0.50, 95% CI: 0.25–0.99, p = 0.0475) (Figure 3).

##### Proprioception

**Figure 4.**
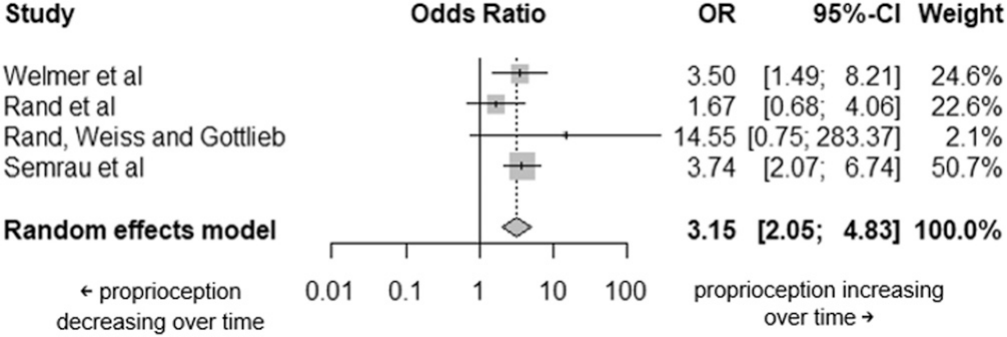
Forest plot of the individual studies included in the proprioception meta-analysis and the overall pooled effect. Smaller values in OR indicate proprioception decreasing over time, and greater values in ORindicate proprioception increasing over time. All studies used for this assessment indicate an improvement in proprioception over time.

There were six studies which assessed proprioception in time, with only four of these providing information in appropriate detail for this meta-analysis. Proprioception measurement tools were a robotic arm position matching task and the Thumb Localisation Test (in one and three studies, respectively). All four studies had an initial assessment timepoint of within 1 week of stroke. The final assessment timepoint was at 6 weeks post-stroke for two studies, six and a half months post-stroke for one study and at 18 months post-stroke for the final study. The four studies included a total of 239 participants, with a total of 19 lost between timepoints. This analysis highlighted that it was more likely to have impaired proprioception at the start, than at the end of the studies i.e. proprioception improves over time ( $I^{2}$ = 10.7%, 95% CI: 0.0%–86.3%; OR = 3.15, 95% CI: 2.05 - 4.83, *p* < 0.0001) (Figure 4).

##### *Motor* Function

**Figure 5.**
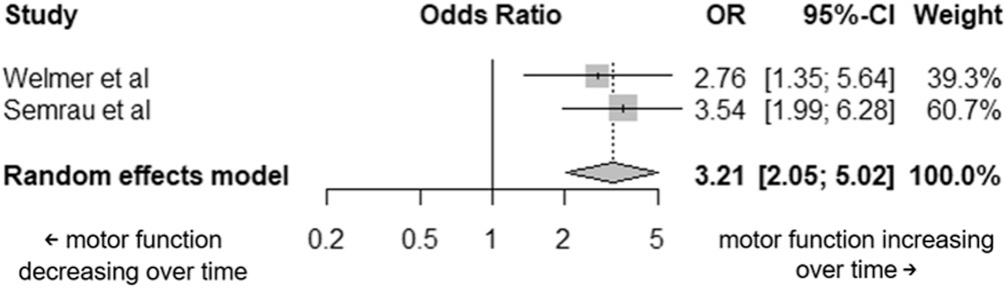
Forest plot of the individual studies included in the motor function meta-analysis and the overall For Peer Review pooled effect.

From the 26 studies that reported different timepoints assessing motor function, only two had sufficient detail in the data reported to be included in this analysis. Motor function assessment tools used were kinematics using robotics in one study and part one of the Lindmark Motor Assessment Scale in the other. Both studies had initial timepoint assessments at a week post-stroke. The final assessments were at six and half months post-stroke for one study and 18 months post-stroke for the other. From the random-effects model, an improvement in motor function in time was highlighted (OR = 3.21, 95% CI: 2.05–5.02, *p* < 0.0001) (Figure 5).

#### Strength of relationships between variables

**Figure 6.**
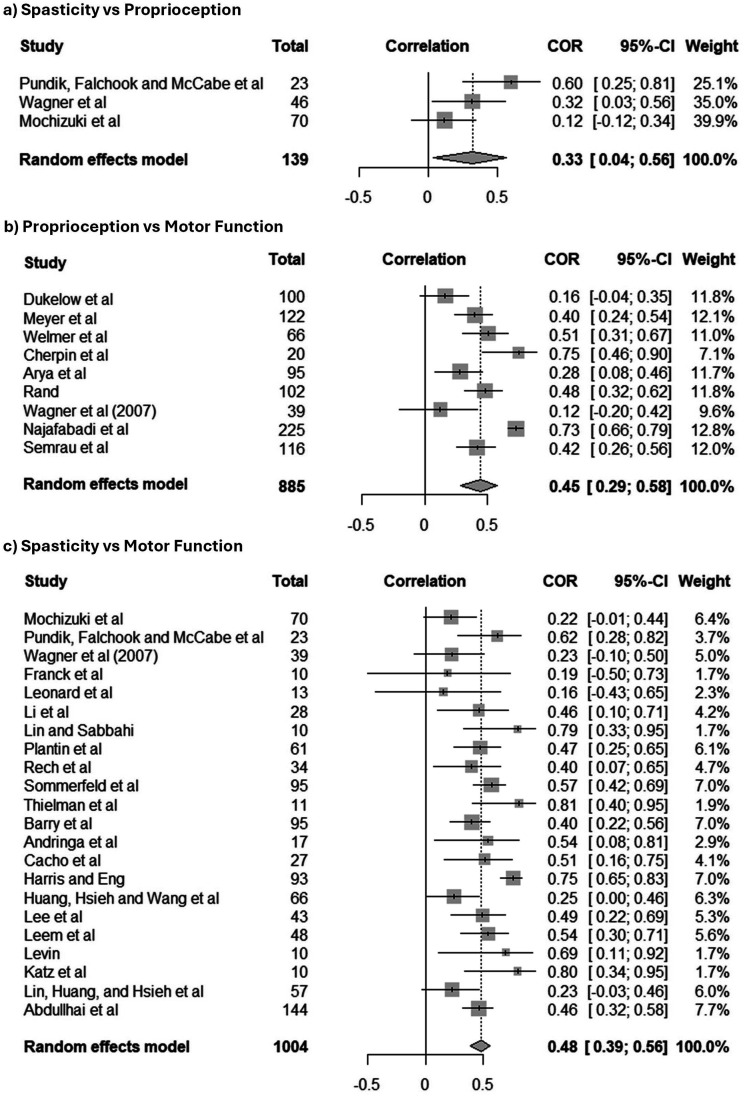
Forest plots of the correlation meta-analyses, (a) spasticity vs proprioception, (b) proprioception vs motor function and (c) spasticity vs motor function.

The random-effects model ( $I^{2}$ = 61.9%, 95% CI: 0.0%–89.1%) indicated a weak but statistically significant correlation between spasticity and proprioception (3 studies with 139 participants; r = 0.33, 95% CI: 0.04 - 0.56, p = 0.0283). This suggests that higher levels of spasticity are associated with poorer proprioception, although the association is modest (Figure 6(a)).

In contrast, the random-effects model ( $I^{2}$ = 87.7%, 95% CI: 78.8%–92.9%) investigating the correlation between proprioception and motor function (as assessed from nine studies and 885 participants) found it was moderate in strength and statistically significant (r = 0.45, 95% CI: 0.29 – 0.58, *p* < 0.0001). This moderate correlation indicates that better proprioception is associated with improved motor function (Figure 6(b)).

Furthermore, from the random-effects model ( $I^{2}$ = 59.2%, 95% CI: 34.7%–74.5%) a moderately strong, statistically significant correlation was observed between spasticity and motor function (22 studies with 104 participants; r = 0.55, 95% CI: 0.46 – 0.62, *p* < 0.0001), suggesting that higher levels of spasticity are associated with poorer motor function (Figure 6(c)). As the number of studies included was more than ten, publication bias could be assessed in the spasticity and motor function analysis. Egger’s regression test indicated that there was no publication bias present in the included studies (t = 0.560, df = 20, *p* = 0.58).

Eight alternative methods to undertake correlations were used across studies. These were: univariate logistic, multivariate logistic and multivariate linear regression, logistic generalized estimating equations, predictive values, chi-squared, paired-t tests and linear mixed modelling. Four other studies reported only *p*-values of the existing correlation. The results of these additional studies could not be included in the meta-analysis of relationships and hence their effect on the overall estimate of existing relationships is not quantified.

## Discussion

This review seems to be the first to report on the relationships between spasticity, proprioception, and motor function of the upper limb post-stroke. The most studied relationship was spasticity and motor function, followed by proprioception and motor function and finally, spasticity and proprioception (in 78.84%, 26.92% and 5.76% of studies, respectively).

Between current statistics regarding the stroke population and the samples from the studies included in this work, differences in demographics can be observed. Some studies included samples younger and some older than the mean age of stroke (68 in men and 73 in women^69^). However, the mean age of the included participants across all 52 included studies was 61 years indicating on average, included participants were younger than what are considered the population means, which may indicate that stroke is becoming more common in younger populations. Furthermore, recent research that highlighted stroke incidence in men aged 20–44 increased over the 22-year period over which the study was carried out.^70^ In addition, over 80% of the included studies involved had more male participants than female, with an overall ratio of female to male participants of 0.713. Yet, women are more at risk of stroke than men.^71^ Findings suggest females may be underrepresented in current stroke research studies and including more female participants in future studies may allow for further investigation into the differences in stroke mechanisms between genders.

Among the included studies, there were also differences in the measurement tool used to assess each variable. In total, eight spasticity, six proprioception and 19 motor function tools were used across all included studies. This meant that comparison between studies was difficult as each tool measured each variable in a slightly different way from the others. In general, a move towards using standardized methods of assessment for each variable would be beneficial for future cross-study analyses. The publication of standardised guidelines for motor function assessment in 2017^72^ is a welcomed step forward, however, there is no evidence regarding recommendations for standardised methods to measure spasticity and proprioception.

Despite differences in measurement tools and populations, meta-analyses regarding the changes in time for each variable and the strength of the relationship between variables were performed. Note that the lack of evidence regarding changes in variables over time in included studies negatively impacts any potential generalisability of results, however, the results of these analyses follow closely with what is expected when a person follows a standard recovery progression.^73–75^

In four out of six of the studies that provided data regarding participants with spasticity, the first assessment timepoint was within 1 week of stroke. In the others, the first assessment occurred at 3 months post-event. The second assessment timepoint was at 3 months post-event for the four studies with an initial assessment within 1 week of stroke and was at 12- or 18-months post-event for the remaining two studies. A review conducted by Sunnerhagen et al.^73^ found that the prevalence of spasticity peaks at one-month post-stroke indicating that increases in prevalence within a study population are likely to occur within the first 4 weeks post-event rather than a later point during recovery. Hence, the two studies with first assessment timepoint 3 months post-event, where little to no change in spasticity prevalence was observed, are in line with what can be expected in the overall progression of stroke recovery^73^. Moreover, the findings of this particular meta-analysis are confounded by the use of the Ashworth Scales by included studies to measure spasticity. These scales are highly subjective and have had their validity as a spasticity measure questioned as they cannot be used to differentiate between spasticity and other causes of increased muscle stiffness^10^.

Considering changes in proprioception over time, in the four studies providing longitudinal data about participants with impaired proprioception, proprioception improved over time in all studies regardless of when assessments occurred. The studies in this analysis assessed participants first within the acute phase of stroke and finally within one and a half to 18 months post-stroke. To investigate changes in proprioception, if any, that occur later in recovery, the minimal evidence available^24^ should be built upon to increase understanding of what occurs during these late phases of stroke. Only two studies provided suitable data regarding motor function changes of participants over time for inclusion in a meta-analysis. From these studies, it was found that motor function improved over time (between 1 week and six and half or 18 months post-stroke). This longitudinal improvement in motor function is echoed by many other studies.^26,27,50,63,64^ Interestingly, no heterogeneity was reported in this analysis, but this is likely due to the limited number of studies included.

The results of the proprioception and motor function meta-analyses suggest that participants in the included studies follow the typical sensorimotor recovery trajectory where the majority functional recovery occurs within the first 6 months after a stroke due to the neuronal reorganization and increased plasticity, coupled with intensive rehabilitation therapies that are given during this period.^74,75^

Shifting focus to the results of the correlation meta-analyses, intriguing insights were noted. To begin, the analyses suggest that spasticity and proprioception are weakly correlated. The involvement of muscle spindles in both spasticity^76^ and proprioception mechanisms^8^ suggests that it is likely some sort of association is present between these variables however, only three studies were included in the meta-analysis of correlation coefficients indicating that there is a severe lack of evidence in this area. This lack of evidence, along with the variation in findings by the individual studies (indicated by the moderate heterogeneity within the meta-analysis) suggests that this relationship needs to be explored further to pinpoint its true nature. Furthermore, the other analyses provided evidence that both spasticity and proprioception are independently moderately correlated with motor function where a decrease in spasticity or improvement in proprioception can lead to an improvement in motor function abilities^17^. The moderate correlations highlight the importance of addressing both spasticity and proprioception in interventions aimed at improving motor function in people who have sustained a stroke.

Within this research, although every effort was made to ensure accurate and valid results, limitations were still present. For example, to perform all meta-analyses, due to the range of measurement tools used, outcomes were grouped under one heading relating to the appropriate variable, regardless of the tool used. For example, the Box and Blocks test which assesses hand dexterity only and the Fugl-Meyer Assessment which assesses a much wider array of motor functions^77^ were both grouped under motor function outcomes. Kinematic measures of upper limb function were also included as motor function outcomes, however, there is a consensus that these should be used to assess the quality of movement rather than aspects of overall function.^78^ This grouping did not only occur with motor function outcomes but also with spasticity and proprioception outcomes and is a limitation of the current analyses as these groupings could have impacted the results obtained.

Additionally, to perform the meta-analyses regarding changes in variables over time, grouping of timepoints had to occur. All baseline measurements were grouped under ‘start’ and all final measurement timepoints, regardless of when they occurred were grouped under ‘end’. Progression of recovery from stroke varies between people^31^ and the longer after stroke a person is, the more time they have had to work on recovery and the more likely it is that recovery may have plateaued.^75^ Due to a lack of evidence in the included studies, sub-analyses of changes between set specific timepoints could not be performed. As more literature is produced regarding longitudinal changes in these recovery outcomes ability post-stroke a meta-analysis looking at set timepoints should be carried out to build upon the limitations of the current analysis. Furthermore, in the current analyses, only the presence of a deficit was assessed rather than the severity of the deficit. There was no consideration of changes in severity over time, however, this would not have been possible due to the level of detail in the data provided.

Three main areas were identified for future research focus. Firstly, it is crucial to quantify the relationships between spasticity and proprioception (assessed in three studies) and between proprioception and motor function (assessed in 14 studies) to obtain a more comprehensive understanding. This should include multivariate analysis to understand the synergy between these variables and their impact on post-stroke recovery. Secondly, assessing participants in the early stages and longitudinally will enable analysis of relationships over time. This could illuminate the poorly understood interactions between early neural recovery and rehabilitation interventions.^74^ Finally, new studies quantifying spasticity should document the prescription of anti-spasticity medication and any changes in participants’ abilities while taking this medication. This documentation would be advantageous and could help explore whether the medication affects any existing association.

## Conclusion

Meta-analyses of currently available evidence revealed moderate correlations between spasticity and motor function, and between proprioception and motor function, while the correlation between spasticity and proprioception was weak. Further research is needed to fully comprehend the interplay between these variables, which could enable the prediction of recovery in one function based on changes in another. Moreover, a comprehensive understanding of these relationships could enhance the personalization of rehabilitation programs, and inform development of new rehabilitation interventions, helping to achieve the most efficient recovery trajectory for each individual after stroke and maximizing their potential for regaining independence.
